# Identifying the Problem Side with Single-Leg Squat and Hamstrings Flexibility for Non-Specific Chronic Low Back Pain

**DOI:** 10.3390/medicina60091428

**Published:** 2024-09-01

**Authors:** Boon Chong Kwok, Helen Elizabeth Smith, Pui Wah Kong

**Affiliations:** 1 Health and Social Sciences (Physiotherapy), Singapore Institute of Technology, Singapore 138683, Singapore; boonchong.kwok@singaporetech.edu.sg; 2 Physical Education and Sports Science Department, National Institute of Education, Nanyang Technological University, Singapore 637616, Singapore; 3 Lee Kong Chian School of Medicine, Nanyang Technological University, Singapore 308232, Singapore; h.e.smith2@keele.ac.uk

**Keywords:** postural sway, motor control, lumbago, movement assessment, DMA Clinical Pilates

## Abstract

*Background and Objectives*: In patients with non-specific chronic low back pain (LBP), their pain and problem sides can differ. Clinical Pilates assessment provides an approach to identify the problem side, but this approach requires experience and can be subjective. This study aimed to investigate if objective measures of single-leg squat postural control and hamstrings flexibility could identify the problem side in adults with non-specific chronic LBP. *Materials and Methods*: Forty adults with non-specific chronic LBP were tested on single-leg squat postural control and hamstrings flexibility. The problem side of participants was assessed with the Clinical Pilates method. Paired *t*-tests were used to compare the postural sway parameters of the single-leg squat and hamstrings flexibility between the problem and non-problem sides. Cohen’s kappa was then used to assess the agreement of postural sway and flexibility measures with the Clinical Pilates method. *Results*: The problem side showed smaller vertical force variance, larger sway path distances, lower peak vertical force, smaller terminal knee flexion angle, longer time to complete the five single-leg squats, and tighter hamstrings as compared to the non-problem side. However, only the overall and anteroposterior sway path distances, terminal knee flexion angle, total squat duration, and hamstrings flexibility yielded moderate to strong agreement with the Clinical Pilates method. *Conclusions*: Single-leg squat postural sway parameters and hamstrings flexibility can objectively identify the problem side in adults with non-specific chronic LBP.

## 1. Introduction

Low back pain is highly prevalent among adults [1], with most people recovering within the first 3 months or requiring pathological treatment strategies [2], but some cases become chronic [3] and are usually non-specific [4]. Although there has been a shift towards a biopsychosocial approach in managing chronic low back pain [5], it is possible that some of the pain experienced by people arises from abnormalities in movement control. Movement control extends beyond specific impairments such as respiratory and spinal muscles, as well as the role of the thoracolumbar fascia [6]. Current evaluations of treatment effects for people with non-specific chronic low back pain rely on subjective patient-reported outcome measures such as pain and function [7,8,9,10]. In view of subjective history taking lacking diagnostic accuracy [11], The Dance Medicine Australia (DMA) Clinical Pilates method uses information about the injuries of an individual such as movements that trigger pain or other symptoms to postulate movement preferences [12]. Traditional Pilates exercises involve movements of the body and limbs in various directions, which is claimed as the best exercise method for chronic low back pain [13,14]. However, there are many variations in Pilates methods, and the lack of descriptions of the methods can limit the adoption of Pilates in clinical practice [15]. A recent study showed that using Pilates exercises as lumbar stabilization exercises was not different from lumbar strengthening exercises in improving pain [16], possibly because both groups had exercise movements that were similar. The DMA Clinical Pilates method categorizes the exercises into different movement patterns to identify patterns that are easy for an individual to perform, also known as movement preference identification [17]. The DMA Clinical Pilates method has evolved from the McKenzie method of lumbar flexion or extension movement preference, where the latter is known to have moderate treatment efficacy for chronic low back pain [18], and integrates the McKenzie method with Pilates exercises. Clinical experience is essential to use the DMA Clinical Pilates method well in accurately identifying the problem side (the side with movement preferences) [17], which may impose challenges to early career therapists. The side with pain may not correspond to the problem side [17]. The lack of knowledge in identifying the problem side could have led to the gap in exercise prescription capability among physiotherapists [19]. However, considering that the DMA Clinical Pilates method requires experience despite training, it would be useful to explore the objective biomechanical measurement method to identify the problem side, thereby complementing existing clinical assessments of patients with non-specific chronic low back pain.

A common motor control assessment is the single-leg squat (SLS) test [20], but it has not been considered in the DMA Clinical Pilates literature [21,22]. Subjective ratings of the SLS quality between assessors can be unreliable [19]. Postural sway measures of the SLS test provides an objective means to evaluate the coordination of the trunk and lower limb muscles to maintain postural stability [23,24,25], but its use for non-specific chronic low back pain is limited to dominant leg evaluation [26]. It is inconclusive which SLS postural sway parameters are useful in guiding clinical diagnosis due to differences in the testing methods between studies [27] and postural control strategies between people with non-specific chronic low back pain [28,29]. Furthermore, postural stability could be influenced by back muscles that are guarding against pain [29], which limits trunk and hip movements, and hence affecting flexibility. Improvement in flexibility after Pilates exercises was found in studies using the sit-and-reach and finger-to-floor methods [30,31]. Improvements in the finger-to-floor method could be attributed primarily to the hamstrings muscle, as lumbar flexion flexibility improvements were not significant [30]. It is possible that some SLS postural sway parameters and hamstrings flexibility could identify the problem side of people with non-specific chronic low back pain.

In view of the current research gaps, there is a need to develop objective methods to support clinical assessment on low back pain. This study aimed to investigate if SLS postural sway parameters and hamstrings flexibility differed between the problem and non-problem sides in individuals with non-specific chronic low back pain, and which objective measurements agreed with the DMA Clinical Pilates assessment. We hypothesized that SLS postural sway parameters and hamstrings flexibility would differentiate the problem side from the non-problem side and that the measurements would agree with the DMA Clinical Pilates assessment. The findings would facilitate clinicians in decision making on the side to prescribe exercises.

## 2. Materials and Methods

### 2.1. Study Design

This study was part of a larger study prospectively registered on the Australian New Zealand Clinical Trials Registry, ACTRN12622001195741. This study was approved by the Institutional Review Boards of the Nanyang Technological University, NTU-IRB-2022-492, and the Singapore Institute of Technology, SIT-IRB-2022150. Participants were tested on one occasion at the university laboratories or clinical sites. Data collection was carried out by the first author (BCK) and assisted by trained research assistants. The DMA Clinical Pilates assessment was performed by the first author (BCK), a physiotherapist with more than 15 years of clinical experience and a certified DMA Clinical Pilates instructor.

### 2.2. Participants

Participants were recruited from universities, private physiotherapy practice and community healthcare centers, and the general public. Forty participants were needed based on an a priori sample size calculation using G*Power version 3.1.9.4 for moderate effect size (0.5), 80% power, α at 5%, and in consideration of a 15% drop-out rate. This study included adults aged 21 to 40 years old who communicated in English, had current pain in the lower back for more than 3 months, with and without lower limb symptoms on most days of the week, and average pain in the past week ≥ 4 points rated on the 11-point pain numeric rating scale [32]. We excluded participants who had spinal surgery or recent treatment for managing low back pain (past 6 months), on-going fever or infection, recent unexplained weight loss, loss of appetite or spinal fractures (past 3 months), malignant cancer or received cancer treatment, cauda equina lesion, complete loss of bladder/bowel control or saddle paresthesia, known pregnancy, or spinal inflammatory disease (ankylosing spondylitis and rheumatoid arthritis). Fifty-eight adults responded to a pre-participation survey; in total, 18 were excluded as 8 no longer had pain, 4 did not meet the age criteria, and 6 were unable to arrange a convenient appointment. Informed, written consent was obtained from each participant.

The participants were first surveyed for their age, gender, ethnicity, dominant leg, pain intensity, functional status with the patient-specific functional scale, disability with the Oswestry Disability Index, psychosocial quality of life with the World Health Organization Well-Being Index, chronicity of low back pain, and physical activity level. The dominant leg was identified with the question, “Which leg would you kick the ball with?” [33]. Participants reported their average pain in the past seven days on an 11-point Likert scale of 0 to 10, where 0 is no pain and 10 is the worst imaginable pain [34]. Participants then provided information on how long they had experienced lower back pain approximated to the nearest month. Next, the participants were interviewed for their weekly physical activity and/or exercise participation based on the existing cut-offs, 150 min for moderate intensity and 75 min for high intensity [35]. Since there was no information for people who participated in a mixture of moderate- and high-intensity activities, we undertook a cautious approach and used 120 min as the cut-off. 

The participants were then interviewed with the patient-specific functional scale [36]. The participants listed one to two routine activities in which they experienced difficulties due to their lower back pain. If two activities were listed, the average of the two ratings was calculated for data analysis. The participants continued to complete the original 10-item Oswestry Disability Index, which included an optional item [37]. The self-reported interview ended with the World Health Organization Well-Being Index 5-item questionnaire [38]. The questions were related to the psychosocial health of the participants over the past two weeks. The total scores of the Oswestry Disability Index and World Health Organization Well-Being Index 5-item questionnaire were each computed to percentages. Upon completion of the interview, the height and weight of each participant was measured using a Seca digital column scale, and then the body mass index was calculated.

### 2.3. Assessments

#### 2.3.1. Single-Leg Squat

**Figure 1:**
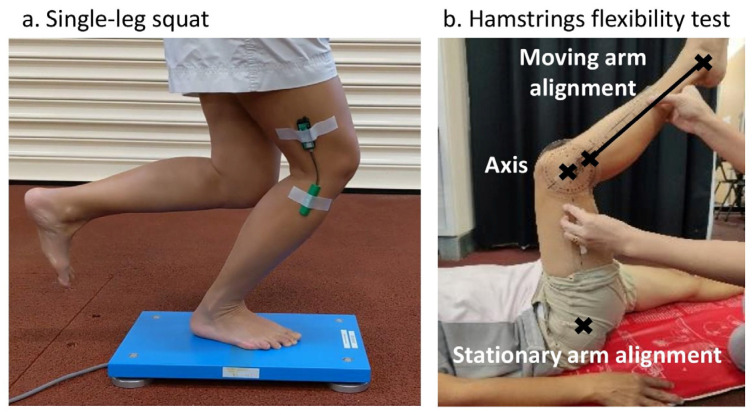
Assessment of (**a**) single-leg squat using a force plate and an electrogoniometer, (**b**) hamstrings flexibility using a goniometer.

Participants performed the SLS test for five continuous squats on a portable force plate (9260AA6, Kistler Instruments, Winterthur, Switzerland) (Figure 1a). The ground reaction force and center of pressure were measured using a force plate (Bioware version 5.4.9) at 1000 Hz for 20 s. The SLS test was not paced with a metronome to study natural performance of the participants. A wireless twin-axis electrogoniometer (Biometrics Ltd., Newport, UK) was secured to the side of the knee to measure the knee flexion angles of the squats (Figure 1a), measured to the nearest 0.1°, with repeated measurements that varied by 0.3° [39]. Due to technical issues with the electrogoniometer for 10 participants, video tracking of the knee flexion angle was used as an alternative. To ensure good consistency, retroreflective markers were placed on the lateral mid-thigh, lateral mid-knee joint, and lateral malleolus of these participants. Their knee movements were videoed at 30 Hz using a mobile phone (Xiaomi Mi 9) mounted on a stable chair from a sagittal view and subsequently analyzed with Kinovea software (version 0.9.5).

The force plate data acquisition was first set to preparation mode, and participants were instructed to step up onto the force plate. Upon standing on the force plate, the participants were instructed to cross their arms on their chest and then to position themselves in a single-leg stance with the non-weightbearing hip slightly flexed and the knee flexed until the tibia was approximately parallel to the ground (Figure 1a). The participants looked straight with a 2-m unblocked view. The electrogoniometer was zeroed, followed by knee flexion angle recording. In the absence of the electrogoniometer, video recording was used. The force plate data acquisition started, and a countdown of 3, 2, 1 was given by the assessor to guide the participants to start the five SLS. Participants were guided to squat with at least 30° knee flexion without losing their balance during the practice trial of five SLS repetitions to familiarize them with the test requirements, which was the same as the actual trial. Upon completion of the fifth squat, participants remained in the single-leg stance position until force measurement was completed. A 30-s seated rest was then given to the participants before the actual trial. Two participants could not complete the actual trial after losing their balance, so their practice trial data were used.

#### 2.3.2. Hamstrings Flexibility Measurements 

The passive knee extension hamstrings flexibility test was performed in the supine position, with the hip and knee flexed to 90 degrees. The assessor passively extended the knee and maintained the hip flexion angle until a firm end-feel was felt, followed by joint angle measurement with a handheld goniometer (Figure 1b) [40]. The stationary axis was the lateral epicondyle of the femur, while the stationary arm was aligned to the greater trochanter of the femur, and the moving arm was aligned to a line formed by the fibular head and lateral malleolus [41]. For each side, the greater angle of two measurements of each leg was recorded. The standard error of measurement using the handheld goniometer measurement for the knee was previously reported to be 4.1° [42].

#### 2.3.3. Clinical Pilates Assessment

The main purpose of the DMA Clinical Pilates assessment in this study was to identify the problem side of the participants who were experiencing non-specific chronic low back pain. The participants’ histories were subjectively assessed to provide provisional directional preference(s) diagnosis using four aspects of physiotherapy practice [17]. The first aspect is the mechanism of injury. For example, a fall on the buttocks into long-sitting position is interpreted as flexion trauma, while landing in the prone position is interpreted as extension trauma. The second aspect is body charting present and past injuries or trauma, where the side with more trauma is hypothesized as the problem side. The third and fourth aspects are inversely related and are easing and aggravating factors. In the instance where sitting slouch eases the pain while sitting upright worsens the pain, flexion preference is hypothesized, and the directional trauma is extension.

**Figure 2:**
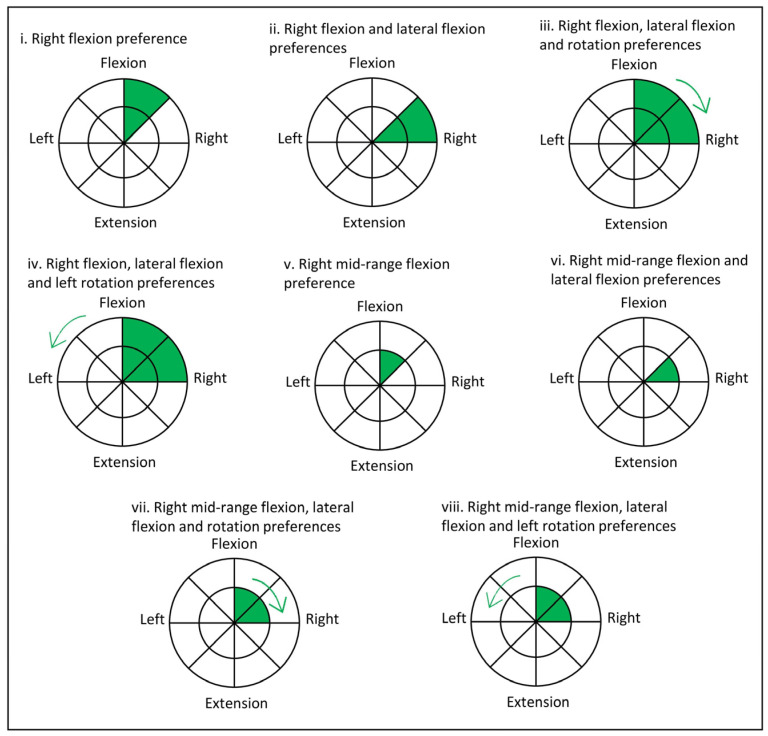
Examples of right flexion directional preference (in green) assessed with the DMA Clinical Pilates method that could find unidirectional (**i**,**v**), duo-directional (**ii**,**vi**), and multi-directional (**iii**,**iv**,**vii**,**viii**) preferences.

The DMA Clinical Pilates exercise testing confirms the hypothesized problem side and directional preference(s) from subjective assessments [12], which can be a combination of movement into either trunk–hip flexion or extension, lateral flexion, and/or rotation. Based on the hypothesis from the aforementioned subjective assessment, a corresponding Pilates exercise in that direction is used to confirm the hypothesis [22]. In the scenario of hypothesized right flexion preference, bug legs or single-leg stretch exercise is used to confirm that moving the right leg will produce better movement quality as compared to moving the left. In the event that the hypothesis cannot be confirmed, the side-lying clamshell exercise is used to test for lateral flexion coupling with flexion, mid-range flexion, mid-range extension, or extension preference on the right [43]. Figure 2 provides examples of the right side as the problem side and different directional preference coupling. The problem side is the same as the side that exhibited directional preference(s). The DMA Clinical Pilates practitioner indicated the problem side using a diagnostic bullseye [17].

### 2.4. Data Processing

**Figure 3:**
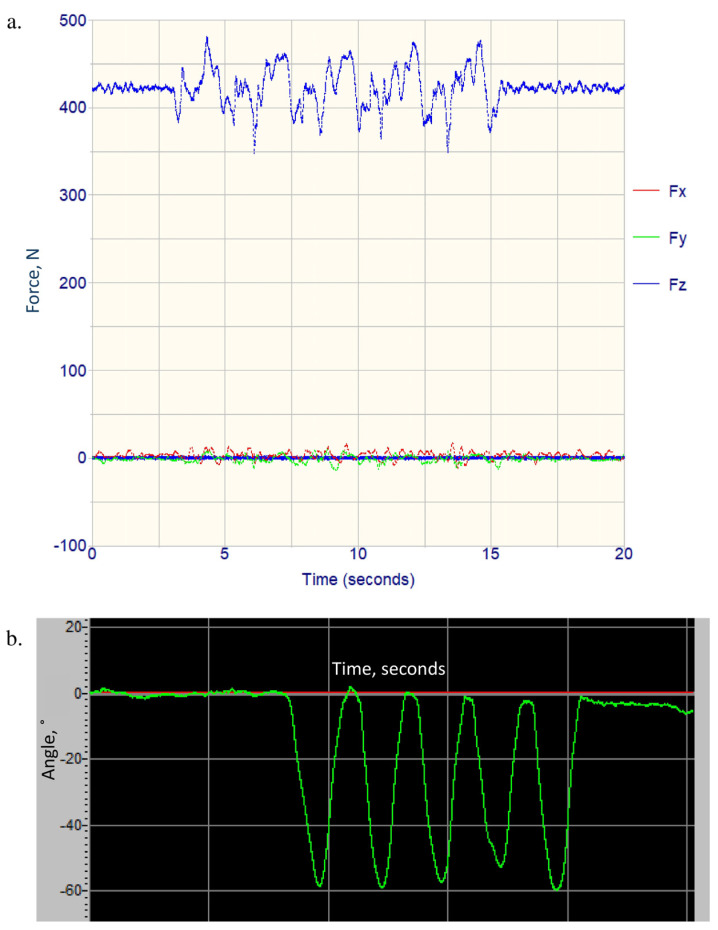
Examples of raw data acquired during the SLS test: (**a**) force plate data collected via the Bioware software, in Newtons (N), and (**b**) knee angle (green line) data collected using the electrogoniometer via the Biometrics software, in degrees.

Examples of raw data collected from the force plate and the electrogoniometer during the SLS test are shown in Figure 3. The ground reaction forces (anteroposterior, mediolateral, and vertical directions) and center of pressure positions (x, y) were exported to a text file using the Bioware software version 5.4.8, with auto-center correction applied. The electrogoniometer raw file was exported using the Biometrics software version 11 for *x*-axis data (knee flexion) with standard ASCII filtering and the engineering units option to a text file. The exported files were then analyzed using a customized MATLAB program (version R2022a). Raw force data were low pass filtered at 50 Hz using a 4th order Butterworth filter. Key postural sway parameters were calculated, including the displacement variances (standard deviation); speed; sway path distances; 95% sway area; variances in the anteroposterior, mediolateral, and vertical forces; and the duration to complete five squats [44]. In addition, the peak vertical force and terminal knee flexion angle of each squat was identified, and the average value of the five squats was calculated. The postural sway variables of the left and right sides were then classified into problem and non-problem sides based on the diagnostic bullseye of each participant, and similar procedures were performed for the hamstrings flexibility measures.

### 2.5. Statistical Analyses

The study population demographics are presented in means (standard deviations) for continuous variables and counts (percentages) for categorical variables. Data normality was assessed using the Quantile-Quantile plot for each parameter of postural sway and hamstrings flexibility. All statistical analyses were performed using IBM SPSS Statistics for Windows, version 23 (IBM Corp., Armonk, NY, USA). Statistical significance was set at *p* < 0.05.

To compare between the problem and non-problem sides, paired sample *t*-tests were used to examine the postural sway parameters and hamstrings flexibility measures. Differences in the SLS postural sway parameters are based on statistical significance, whereas for hamstrings flexibility, differences should exceed 4.1° in addition to statistical significance. Data are expressed as the means (standard deviations) with 95% confidence intervals. The postural sway and flexibility measures that identified the problem side were further examined for agreement with the DMA Clinical Pilates method using Cohen’s kappa (κ) coefficient. A 0.4 cut-off was used to indicate moderate agreement and 0.6 for strong agreement [45].

## 3. Results

The participants’ physical and demographic characteristics are presented in Table 1. All participants completed the study without adverse events or missing data. Fewer than half of the participants were found to have their pain side corresponding to the problem side, *n* = 17 (Table 2). This shows that body charting the pain side does not indicate the problem side in more than 50% of the participants.

Half of the postural sway parameters yielded statistically significant differences between the problem and non-problem sides (Table 3). The problem side swayed more than the non-problem side for overall, anteroposterior, and mediolateral sway paths. The problem side required more time to complete the five squats, with a lower average peak vertical force and, correspondingly, a smaller terminal knee flexion angle during the squats as compared to the non-problem side. The hamstrings muscle flexibility was lower in the problem side as compared to the problem side. The agreement between the overall sway path distance, anteroposterior sway path distance, average terminal knee flexion angle, overall squat duration, and hamstrings flexibility for problem side identification referenced against the DMA Clinical Pilates method ranged from moderate to strong as shown in Table 4.

## 4. Discussion

The present study investigated the use of the SLS postural sway and hamstrings flexibility in identifying the problem side of adults with non-specific chronic low back pain. The findings indicated that the problem side could be identified by seven postural sway parameters, but only four had moderate agreement with the DMA Clinical Pilates method. Hamstrings flexibility successfully identified the problem side and agreed strongly with the DMA Clinical Pilates method. This study included a diverse adult population with varying levels of physical activity (general exercise to sports) participation. The participants were 20% above the cut-off for risk of clinical depression [38]; thus, they were unlikely to have psychosocial issues. The findings suggest that objective measures of SLS postural sway and hamstrings muscle flexibility could complement existing clinical practice for the assessment of non-specific chronic low back pain.

### 4.1. Single-Leg Squat Postural Sway

A previous study was unable to identify useful SLS postural sway parameters to guide pathological diagnosis of knee pain [27]. In our study on non-specific chronic low back pain, we found that the problem side swayed longer distances (sway paths) as compared to the non-problem side, but not the postural sway area. It has been suggested that for non-specific chronic low back pain, the problem side has impaired motor control [46], for which our study showed smaller range of movements (terminal knee flexion angle) on the problem side. This is most likely a strategy to minimize balance perturbation, translating to smaller vertical force variance and a lower average peak vertical force to exert when transitioning into standing. The latency in squats completion on the problem side reflected poor motor control.

It should be noted that only four out of seven identified postural sway parameters agreed well with the DMA Clinical Pilates method in identifying the problem side. The sway path distance, particularly anteroposterior, was longer in the problem side despite lesser perturbation (smaller average knee flexion angle) as compared to the non-problem side. The DMA Clinical Pilates method predominantly uses front and back movement patterns for the assessment of instability [17], which might explain the sensitivity in anteroposterior sway path distance. The overall squat duration was subtly different between the problem and non-problem sides but sufficient to differentiate the sides. Thus, physiotherapists could evaluate the motor control of patients with chronic low back pain using the SLS test to identify the problem side and to prescribe exercises for this side. This is an important observation because not all physiotherapists are trained in the DMA Clinical Pilates exercise testing method to confirm the side to treat. Physiotherapists would then be able to prescribe specific exercises on the problem side instead of general exercises on both sides. For instance, based on history taking, if the easing factors suggest that sitting and slouching minimize pain and are comfortable positions, while standing and walking aggravate pain or increase discomfort, exercises based on the McKenzie method would suggest treating into flexion [18]. Complemented by information from the SLS test, specific one-sided exercises such as leg slides or front kicks could be prescribed instead of general central abdominal curl-ups.

### 4.2. Hamstrings Flexibility

In our participants with non-specific chronic low back pain, their problem side was found to have tighter hamstrings muscles than the non-problem side (Table 3). Using hamstrings flexibility as a method to identify the problem side corresponded well with the DMA Clinical Pilates method as indicated by the highest κ of 0.67 among all objective measures (Table 4). The strong agreement between hamstrings flexibility and the DMA Clinical Pilates method can be attributed to the same assessor conducting all tests in the present study. However, the 3.9° difference in hamstrings flexibility found in our study was slightly lower than the cut-off value of 4.1° for meaningful interpretation. It is uncertain whether similar results can be obtained from other assessors, especially those with less clinical experience.

In a study on the intra-rater reliability of several lower limb flexibility measures, variability in repeated measures ranged from 5° to 7° [47]. Specific to our method of passive knee extension, the variability in measurements among different assessors averaged 7.6° [48]. The differences in hamstrings flexibility measures can be due to the varying force exertion by the assessors and/or the experience of the assessor in determining the firm end-feel. To this end, the SLS test may have the advantage over hamstrings flexibility measures because the biomechanical analysis of postural sway measures does not depend on the testers’ clinical experience.

### 4.3. Limitations

This study has a few limitations. First, we studied young adults, from 21 to 40 years old, so generalizing this study’s findings to older age groups requires careful consideration. Future studies can consider studying older age groups that may present with poor postural stability during the SLS test. Next, our study investigated SLS postural sway and hamstrings flexibility, but there are other measures that can complement the clinical assessments of low back pain. For example, the Lift and Place (LAP) test has shown promising results in assessing lifting disability in individuals with chronic low back pain [49].

## 5. Conclusions

This study identified a few postural sway parameters and hamstrings flexibility that could assist in the identification of the problem side among adults with non-specific chronic low back pain. The postural sway path distances (overall and anteroposterior), average terminal knee flexion angle and squat duration of the SLS test, as well as hamstrings flexibility, could identify the problem side and were in agreement with the DMA Clinical Pilates method. If one is not familiar with the DMA Clinical Pilates method, these objective measures could assist clinicians in identifying the problem side of patients and prescribing exercises on the correct side to maximize the benefit of exercise treatment.

## Figures and Tables

**Table 1:** Study characteristics (*n* = 40).

Demographics	Distribution
Age, years, mean (SD)	32.1 (7.3)
Gender, male, *n* (%)	25 (62.5)
Ethnicity, Chinese, *n* (%)	28 (70)
Dominant leg, right, *n* (%)	36 (90)
Height, m, mean (SD)	1.70 (0.07)
Body mass, kg, mean (SD)	68.5 (12.9)
Body mass index, kg/m^2^, mean (SD)	23.66 (3.52)
Pain visual numeric scale, /10, mean (SD)	4.6 (0.8)
Patient-specific functional scale, /10, mean (SD)	5.9 (1.5)
Oswestry disability index, %, mean (SD)	14 (4.5)
WHO-5, %, mean (SD)	74.2 (8.9)
Chronicity of low back pain, years, mean (SD)	4.37 (0.57)
Physical activity level, *n* (%)	
Does not meet recommendation	11 (27.5)
Meets 150 min/week moderate intensity	18 (45)
Meets 120 min/week moderate and high intensity	8 (20)
Meets 75 min/week high intensity	3 (7.5)
Directional preferences	
Flexion, extension or nil:	
Flexion, *n* (%)	14 (35)
Extension, *n* (%)	24 (60)
Nil, *n* (%)	2 (5)
Lateral flexion present, *n* (%)	31 (77.5)
Rotation present, *n* (%)	10 (25)

WHO-5: World Health Organization Well-Being Index.

**Table 2:** Cross-tabulation of pain and problem sides (*n* = 40).

		Problem Side	
		Left	Right
Pain side (s)	Left	6	3
	Right	3	11
	Both	1	12
	Central	2	2

**Table 3:** Comparison between problem and non-problem sides for objective postural sway and hamstrings flexibility measures, *n* = 40.

Measurements	Sides, Mean (SD)		Mean Difference (SD)	95% CI	*p*-Value
	Problem *	Non-Problem			
Postural sway parameters					
Anteroposterior displacement variance, cm	1.82 (0.54)	1.76 (0.47)	0.06 (0.48)	−0.09 to 0.22	0.410
Mediolateral displacement variance, cm	0.89 (0.23)	0.86 (0.2)	0.03 (0.2)	−0.03 to 0.1	0.341
Anteroposterior speed, cm/s	4.93 (1.17)	4.95 (1.06)	−0.02 (0.3)	−0.12 to 0.07	0.656
Mediolateral speed, cm/s	3.9 (1.32)	3.8 (1.31)	0.1 (0.34)	−0.01 to 0.21	0.077
Anteroposterior force variance, N	9.09 (3.69)	9.2 (2.82)	−0.11 (2.15)	−0.8 to 0.57	0.739
Mediolateral force variance, N	6.44 (2.57)	6.24 (2.25)	0.2 (1.79)	−0.37 to 0.77	0.486
Vertical force variance, N	60.21 (38.91)	71.75 (43.66)	−11.54 (15.9)	−16.63 to −6.46	**<0.001**
Overall sway path, m	190.3 (55.07)	169.04 (44.4)	21.26 (52.55)	4.46 to 38.07	**0.014**
Anteroposterior sway path, m	159.28 (51.08)	141.56 (42.62)	17.72 (54.15)	0.4 to 35.04	**0.045**
Mediolateral sway path, m	76.9 (23.98)	68.13 (21.41)	8.77 (23.23)	1.34 to 16.2	**0.022**
Centre of pressure 95% sway area, cm^2^	311.47 (138.99)	280.72 (86.6)	30.75 (111.07)	−4.77 to 66.27	0.088
Average peak vertical force of 5 squats, N	801.92 (178.02)	812.88 (178.13)	−10.96 (24.85)	−18.91 to −3.01	**0.008**
Average terminal knee flexion angle of 5 squats, degrees	47.59 (11.92)	53.94 (13.53)	−6.35 (9.55)	−9.41 to −3.3	**<0.001**
Overall squat duration, s	10.76 (2.37)	9.86 (2.47)	0.9 (1.49)	0.42 to 1.38	**<0.001**
Hamstrings flexibility, °	136.3 (6.4)	140.2 (6)	−3.9 (5.3)	−5.61 to −2.19	**<0.001**

* Problem side is similar to side of directional preference. Bold font indicates statistical significance (*p* < 0.05).

**Table 4:** Problem side classification by objective measures against the DMA Clinical Pilates method.

Objective Measures		DMA Clinical Pilates		Cohen’s Kappa	
	Problem Side	Left	Right	κ	*p*-Value
Vertical force variance	Left	6	4	0.38	0.017
	Right	6	24		
Overall sway path	Left	12	11	0.48	<0.001
	Right	0	17		
Anteroposterior sway path	Left	11	11	0.42	0.002
	Right	1	17		
Mediolateral sway path	Left	7	8	0.28	0.075
	Right	5	20		
Average peak vertical force of 5 squats	Left	6	10	0.13	0.398
	Right	6	18		
Average terminal knee flexion angle of 5 squats	Left	9	7	0.46	0.003
	Right	3	21		
Overall squat duration *	Left	6	3	0.42	0.008
	Right	6	24		
Hamstrings flexibility ^†^	Left	11	1	0.67	<0.001
	Right	5	22		

* One participant had similar overall squat duration on both sides and was excluded from analysis.^†^ One participant had similar hamstrings muscle flexibility on both sides and was excluded from analysis.

## Data Availability

Data are available immediately following publication (no end date) at the NIE Data Repository (https://doi.org/10.25340/R4/V5VNV3).
